# Supplementary material for: Conserved features of TERT promoter duplications reveal an activation mechanism that mimics hotspot mutations in cancer
Source: Nat Commun. 2022 Sep 16;13:5430. doi: 10.1038/s41467-022-33099-x (PMC9481613; doi:10.1038/s41467-022-33099-x)
Supplement: Supplementary file 1 — Supplementary Information [file 41467_2022_33099_MOESM1_ESM.pdf]

Conserved features of TERT promoter duplications reveal an activation mechanism that mimics hotspot mutations in cancer

SUPPLEMENTARY FIGURES AND FIGURE LEGENDS

Supplementary Figure 1

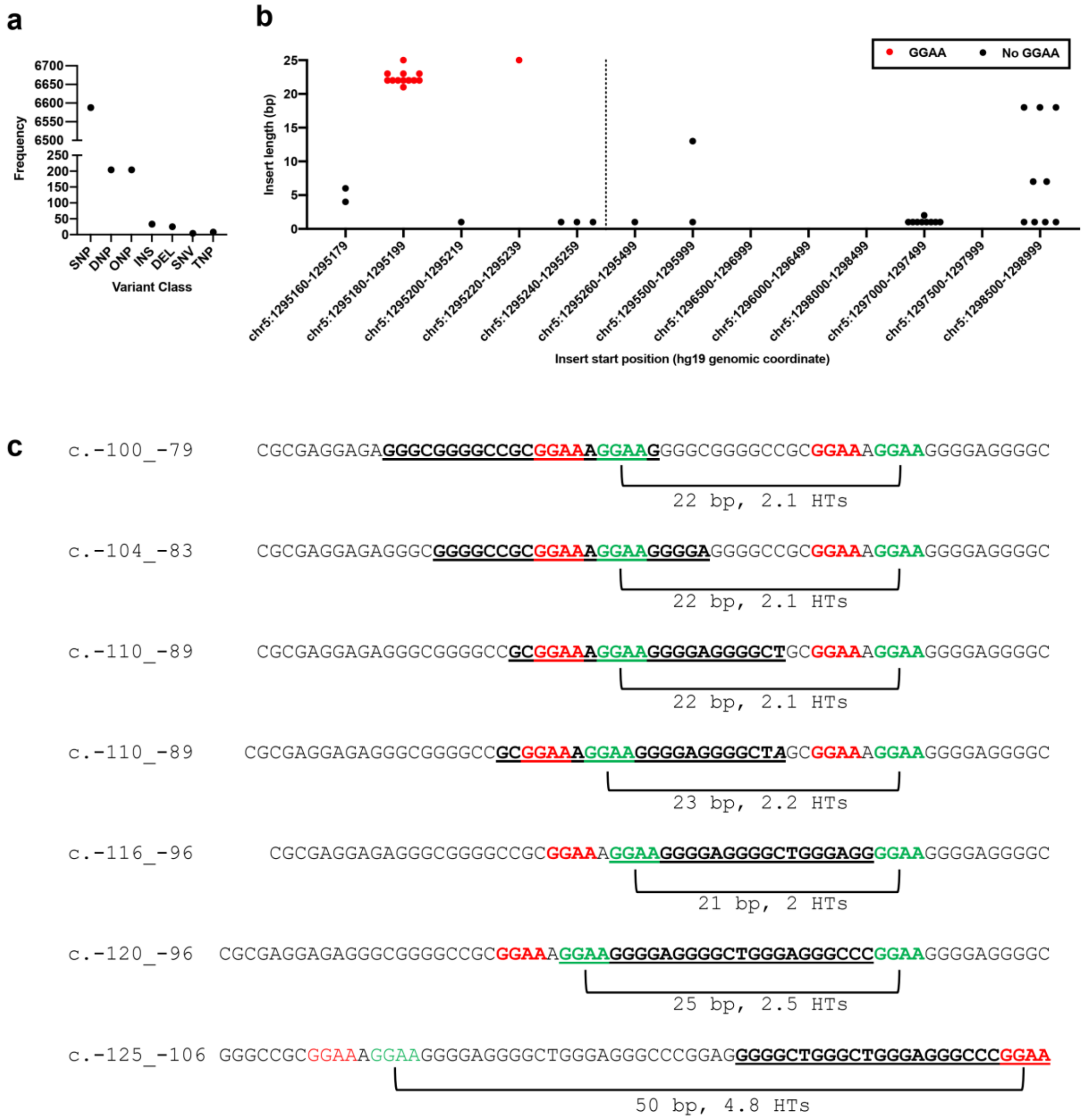

Supplementary Figure 1. Spectrum and frequency of *TERT* promoter variant classes in cancer. (a) Frequency of *TERT*p variant classes in cancer (GENIE, v10 cohort). DEL (deletion), INS (insertion), SNP (single nucleotide polymorphism), DNP (di-nucleotide polymorphism), TNP (tri-nucleotide polymorphism), and ONP (oligo-nucleotide polymorphism for four or more consecutive nucleotides) **(b)** *TERT*p insertion length plotted by hg19 genomic

insert start position (GENIE, v10 cohort). Insertions containing a GGAA motif leading to an ETS transcription factor binding sites are shown in red. Vertical line demarcates a change in the genomic position grouping range. **(c)** Mapping of the *TERT*p duplications from Table 1. Duplications are in bold and underlined. The red text indicates the ETS 195 motif and green text indicates the ETS 200 motif. The distance between *de novo* ETS 200 and native ETS 200 is shown, with the exception of the hotspot duplication (c.-125_-106) where the distance between the *de novo* hotspot duplication and native ETS 200 is shown. Base pair (bp). Helical turns (HTs). Source data are provided as a Source Data file.

Supplementary Figure 2

a

Duplicated Region	Between ETS bp	HTs	Duplication De Novo ETS / Native ETS
-111_-79	33	3.1	**GGGCGGGGCCGCGGAAAGGAAGGGGAGGGGCTG**GGGCGGGGCCGCGGAAAGGAAGGGGAGGGGCTG
-110_-79	32	3.0	**GGGCGGGGCCGCGGAAAGGAAGGGGAGGGGCT**GGGCGGGGCCGCGGAAAGGAAGGGGAGGGGCTG
-109_-79	31	3.0	**GGGCGGGGCCGCGGAAAGGAAGGGGAGGGGC**GGGCGGGGCCGCGGAAAGGAAGGGGAGGGGCTG
-108_-79	30	2.9	**GGGCGGGGCCGCGGAAAGGAAGGGGAGGGG**GGGCGGGGCCGCGGAAAGGAAGGGGAGGGGCTG
-106_-79	28	2.7	**GGGCGGGGCCGCGGAAAGGAAGGGGAGG**GGGCGGGGCCGCGGAAAGGAAGGGGAGGGGCTG
-104_-79	26	2.5	**GGGCGGGGCCGCGGAAAGGAAGGGGA**GGGCGGGGCCGCGGAAAGGAAGGGGAGGGGCTG
-102_-79	24	2.3	**GGGCGGGGCCGCGGAAAGGAAGGG**GGGCGGGGCCGCGGAAAGGAAGGGGAGGGGCTG
**-100_-79**	22	2.1	**GGGCGGGGCCGCGGAAAGGAAG**GGGCGGGGCCGCGGAAAGGAAGGGGAGGGGCTG
-98_-79	20	1.9	**GGGCGGGGCCGCGGAAAGGA**GGGCGGGGCCGCGGAAAGGAAGGGGAGGGGCTG
-96_-79	18	1.7	**GGGCGGGGCCGCGGAAAG**GGGCGGGGCCGCGGAAAGGAAGGGGAGGGGCTG
-94_-79	16	1.5	**GGGCGGGGCCGCGGAA**GGGCGGGGCCGCGGAAAGGAAGGGGAGGGGCTG
-92_-79	14	1.3	**GGGCGGGGCCGCGG**GGGCGGGGCCGCGGAAAGGAAGGGGAGGGGCTG

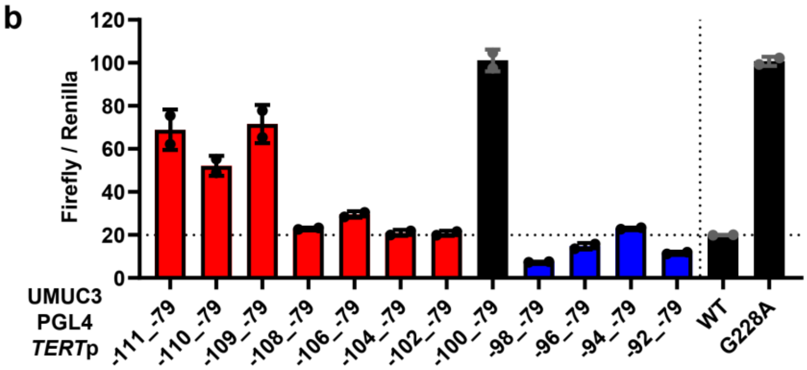

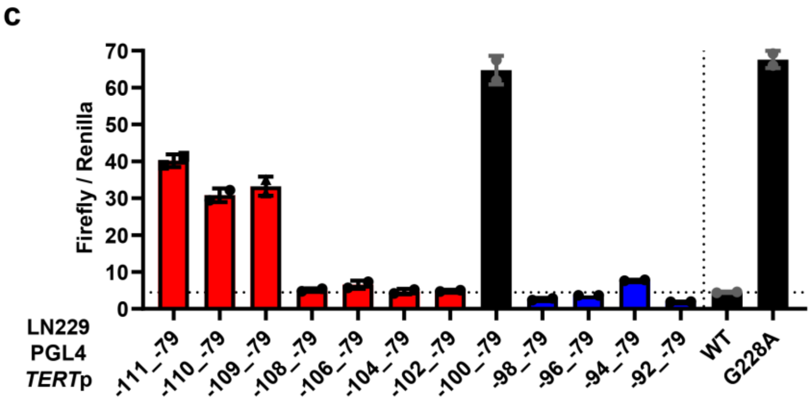

Supplementary Figure 2. *TERT* promoter activation requires *de novo* and native ETS sites in phase. **(a)** Insertion and deletion variants of c.-100_-79 *TERT*p duplication. Duplications are in bold. The red text indicates the ETS 195 motif and green text indicates the ETS 200 motif. The distance (bp) and helical turns (HT) between *de novo* and native ETS is shown. **(b)** *TERT*p luciferase reporter assays for wild type, hotspot mutants and c-100_-79 duplication variants in UMUC3 cells and **(c)** in LN229 cells. Values are mean and SD. The black bars represent the constructs that are positive and negative controls along with the duplication identified in several tumor samples. The blue bars and red bars indicate constructs

for which the ETS sites are moved out of phase and closer together (blue) or further apart (red) compared to the duplication sample. Abbreviations: *TERT*p, *TERT* promoter; WT, wildtype. N=2 independent experiments. Source data are provided as a Source Data file.

Supplementary Figure 3

a

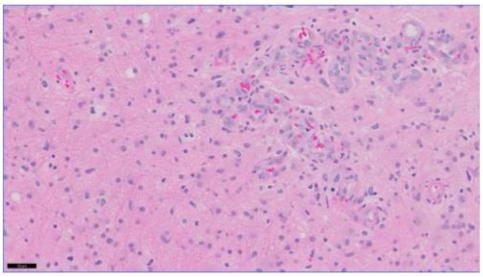

b

PATHOGENIC AND LIKELY PATHOGENIC ALTERATIONS				
VARIANT	TRANSCRIPT ID	CLASSIFICATION	READS	MUTANT ALLELE FREQUENCY
CDKN2A, CDKN2B homozygous deletion	all	Pathogenic	N/A	N/A
EGFR high level amplification	all	Pathogenic	~35,000 (>50x)	N/A
TERT c.-100_-79dupCTTCCTTTCCGCGGCCCCGCCC	NM_198253.2	Likely Pathogenic	593	37%

c

PATHOGENIC AND LIKELY PATHOGENIC ALTERATIONS				
VARIANT	TRANSCRIPT ID	CLASSIFICATION	READS	MUTANT ALLELE FREQUENCY
Trisomy 7, Monosomy 10	N/A	Pathogenic	N/A	N/A
CDKN2A, CDKN2B homozygous deletion	all	Pathogenic	N/A	N/A
ERRFI1 homozygous deletion	all	Pathogenic	N/A	N/A
NF1 p.Y2285*	NM_001042492.2	Pathogenic	851	75%
PTPN11 p.G503A	NM_002834.3	Pathogenic	661	39%
TERT c.-100_-79dupCTTCCTTTCCGCGGCCCCGCCC	NM_198253.2	Likely Pathogenic	1803	27%

d

PATHOGENIC AND LIKELY PATHOGENIC ALTERATIONS				
VARIANT	TRANSCRIPT ID	CLASSIFICATION	READS	MUTANT ALLELE FREQUENCY
Trisomy 7, Monosomy 10	N/A	Pathogenic	N/A	N/A
PTEN exon 4 tandem duplication expected to cause p.V85fs	NM_000314	Pathogenic	44 over duplication junctions	~10%
TERT c.-100_-79dupCTTCCTTTCCGCGGCCCCGCCC	NM_198253.2	Likely Pathogenic	1024	10%
**Sensitivity for Variant Detection Decreased Due to Low Tumor Content**				
The tumor material submitted for testing contained approximately 20.0% neoplastic cells, below the minimum requirement of 25%, which limits the sensitivity of this assay for detection of single nucleotide variants, small insertion/deletion variants, copy number changes, and structural variants..				

Supplementary Figure 3. Histological and molecular features of a glioblastoma with the *TERT* promoter duplication (SF12747/SF13241). (a) H&E staining of FFPE sections from IDH wildtype *TERT*p duplication glioblastoma, recurrent tumor (SF13241). Scale bar denotes 50µm. **(b) (c) (d)** Pathogenic alterations detected with the UCSF500 clinical sequencing panel from IDH wildtype *TERT*p duplication glioblastoma primary tumors (SF12747) from the occipital lobe, parietal lobe and matched recurrent tumor (SF13241), respectively.

Supplementary Figure 4

a

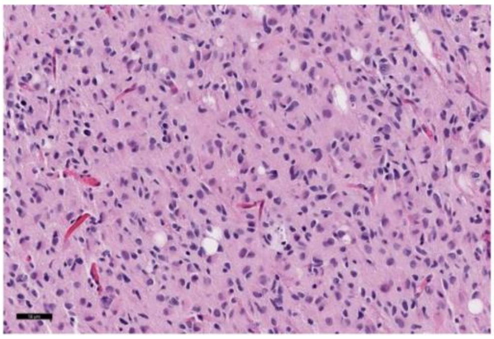

b

PATHOGENIC AND LIKELY PATHOGENIC ALTERATIONS				
VARIANT	TRANSCRIPT ID	CLASSIFICATION	READS	MUTANT ALLELE FREQUENCY
Trisomy 7, Monosomy 10	N/A	Pathogenic	N/A	N/A
CDKN2A homozygous deletion	all	Pathogenic	N/A	N/A
NF1 p.Y2285*	NM_001042492.2	Pathogenic	386	60%
TERT c.-100_-79dupCTTCCTTTCCGCGGCCCCGCCC	NM_198253.2	Possibly Pathogenic	368	39%

Supplementary Figure 4. Histological and molecular features of a glioblastoma with the *TERT* promoter duplication (SF5681). (a) H&E staining of FFPE section from IDH wildtype *TERT*p duplication glioblastoma (SF5681). Scale bar denotes 50µm. **(b)** Pathogenic alterations detected with the UCSF500 clinical sequencing panel from IDH wildtype *TERT*p duplication glioblastoma (SF5681).

Supplementary Figure 5

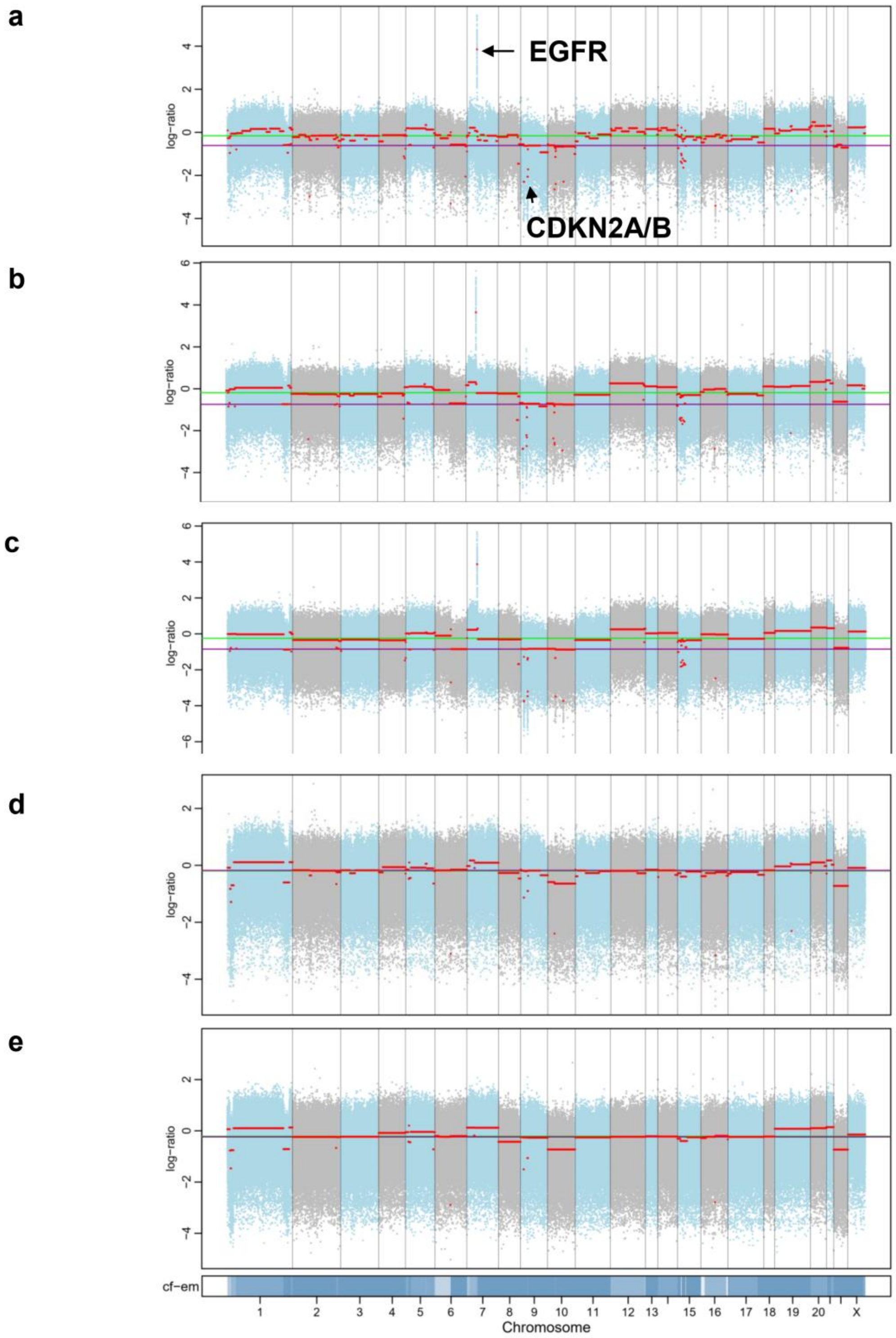

Supplementary Figure 5. Copy number profiles of the primary multifocal glioblastoma with the *TERT* promoter duplication (a-e) Copy number profiles from FACETS analysis of exome-sequencing of different regions of the tumor (SF12747). The tumor has *EGFR*

amplification and *CDKN2A* deletion in C1-C3 (occipital portion), two common alterations in glioblastoma. *EGFR* amplification is absent from D3 and D4 (parietal portion). The *TERT*p duplication is present in all five of these samples (see figure 4g).

Supplementary Figure 6

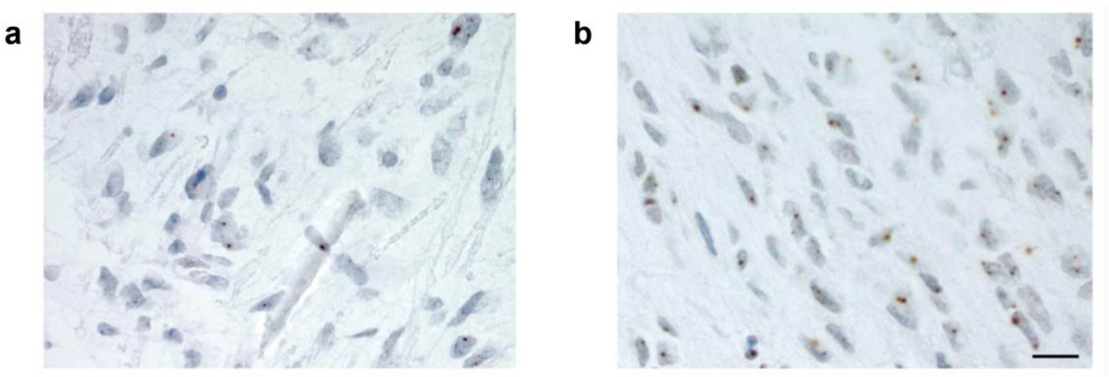

Supplementary Figure 6. Similar *TERT* expression in a glioblastoma with *TERT* promoter duplication compared to a glioblastoma with a mutation. (a-b) RNAscope for *TERT* expression in human primary glioblastoma. Representative images from RNAscope demonstrating **(a)** *TERT* mRNA in a glioblastoma, IDH-wildtype with *TERT* c.-124C>T mutation, **(b)** *TERT* mRNA in an *IDH*-wild type glioblastoma with *TERT* c.-100_-79 duplication (SF5681). Nuclei stained with hematoxylin. Scale bar denotes 30 µm.

SUPPLEMENTARY TABLES

Supplementary Table 1. Number of patients diagnosed with either TERTp hotspot mutation or duplication within all patients diagnosed with glioblastoma in the UCSF500 and GENIE data sets.

Dataset	Glioblastoma (patients)	pathogenic TERTp mutation	TERTp dup	hotspot G228A	hotspot G250A
**UCSF500**	615	449 (73.01%)	2 (0.53%)	275 (72.75%)	101 (26.72%)
**GENIE**	4209	1354 (32.17%)	2 (0.15%)	1049 (77.47%)	303 (22.38%)
