# Supplementary material for: Conserved features of TERT promoter duplications reveal an activation mechanism that mimics hotspot mutations in cancer
Source: Nat Commun. 2022 Sep 16;13:5430. doi: 10.1038/s41467-022-33099-x (PMC9481613; doi:10.1038/s41467-022-33099-x)
Supplement: Supplementary file 3 — Reporting Summary [file 41467_2022_33099_MOESM3_ESM.pdf]

nature portfolio

Corresponding author(s): Joseph F. Costello

Last updated by author(s): Aug 29, 2022

Reporting Summary

Nature Portfolio wishes to improve the reproducibility of the work that we publish. This form provides structure for consistency and transparency in reporting. For further information on Nature Portfolio policies, see our Editorial Policies and the Editorial Policy Checklist.

Statistics

For all statistical analyses, confirm that the following items are present in the figure legend, table legend, main text, or Methods section.

n/a	Confirmed	
☒	☐	The exact sample size (*n*) for each experimental group/condition, given as a discrete number and unit of measurement
☒	☐	A statement on whether measurements were taken from distinct samples or whether the same sample was measured repeatedly
☐	☒	The statistical test(s) used AND whether they are one- or two-sided<br>*Only common tests should be described solely by name; describe more complex techniques in the Methods section.*
☒	☐	A description of all covariates tested
☐	☒	A description of any assumptions or corrections, such as tests of normality and adjustment for multiple comparisons
☐	☒	A full description of the statistical parameters including central tendency (e.g. means) or other basic estimates (e.g. regression coefficient) AND variation (e.g. standard deviation) or associated estimates of uncertainty (e.g. confidence intervals)
☐	☒	For null hypothesis testing, the test statistic (e.g. *F*, *t*, *r*) with confidence intervals, effect sizes, degrees of freedom and *P* value noted<br>*Give P values as exact values whenever suitable.*
☒	☐	For Bayesian analysis, information on the choice of priors and Markov chain Monte Carlo settings
☒	☐	For hierarchical and complex designs, identification of the appropriate level for tests and full reporting of outcomes
☒	☐	Estimates of effect sizes (e.g. Cohen's *d*, Pearson's *r*), indicating how they were calculated

Our web collection on statistics for biologists contains articles on many of the points above.

Software and code

Policy information about availability of computer code

Data collection	CNVkit, Nexus Copy Number (Biodiscovery), FACETS
Data analysis	R, Burrows-Wheeler aligner (BWA), Genome Analysis Toolkit (GATK), Picard CalculateHsMetrics , Picard CollectInsertSizeMetrics, FreeBayes, Unified Genotyper, Pindel, Delly, Annovar, IGV, PyClone (version PyClone-0.13.1), QuPath 0.2.3, ImageQuant, GraphPad Prism v9

For manuscripts utilizing custom algorithms or software that are central to the research but not yet described in published literature, software must be made available to editors and reviewers. We strongly encourage code deposition in a community repository (e.g. GitHub). See the Nature Portfolio guidelines for submitting code & software for further information.

Data

Policy information about availability of data

All manuscripts must include a data availability statement. This statement should provide the following information, where applicable:

- Accession codes, unique identifiers, or web links for publicly available datasets
- A description of any restrictions on data availability
- For clinical datasets or third party data, please ensure that the statement adheres to our policy

The raw exome sequencing data generated in this study have been deposited in the European Genome-Phenome Archive database under accession code EGAD00001008768 [https://ega-archive.org/datasets/EGAD00001008768]. The raw panel sequencing data generated in this study have been deposited in the European Genome-Phenome Archive database under accession code EGAD00001009286 [https://ega-archive.org/datasets/ EGAD00001009286]. The exome and panel sequencing data are available under restricted access for IRB requirements, access can be obtained by contacting J.F.C. (joseph.costello@ucsf.edu). The GENIE dataset can be accessed via https://genie.cbioportal.org/login.jsp after registration on the platform. The data from Panebianco et al. (reference 22) are available from the corresponding author upon reasonable request (we used the processed and published data). Pierini et al. (reference 23) used TERT promoter specific PCR

and Sanger sequencing and thus the data (sequencing traces) and results are available in their publication. Source data are provided with this paper (including uncropped gels and blots).

Field-specific reporting

Please select the one below that is the best fit for your research. If you are not sure, read the appropriate sections before making your selection.

☒ Life sciences ☐ Behavioural & social sciences ☐ Ecological, evolutionary & environmental sciences

For a reference copy of the document with all sections, see nature.com/documents/nr-reporting-summary-flat.pdf

Life sciences study design

All studies must disclose on these points even when the disclosure is negative.

Sample size	Analysis was not performed to determine sample size for the in vitro data. Sample sizes for exome sequencing and qPCR were chosen based on availability of suitable material. Multiple samples from one patient were extracted for valid representation of the whole tumor. RNA scope and EMSA analysis were performed with suitable controls from TERTp mutant and wildtype tumor samples. For the reporter assay two different cell lines were chosen to exclude intercellular variabiliy.
Data exclusions	No data were excluded from the analyses.
Replication	All experiment were done in at least duplicates and compared using two different cell lines. All attempts at replication were successful.
Randomization	Randomization was not applicaple to this study as preexisting conditions (duplication vs hotspot mutation) were tested against each other.
Blinding	Blinding was not applicaple to this study as preexisting conditions (duplication vs hotspot mutation) were tested against each other.

Reporting for specific materials, systems and methods

We require information from authors about some types of materials, experimental systems and methods used in many studies. Here, indicate whether each material, system or method listed is relevant to your study. If you are not sure if a list item applies to your research, read the appropriate section before selecting a response.

Materials & experimental systems

n/a	Involved in the study	
☒	☐	Antibodies
☐	☒	Eukaryotic cell lines
☒	☐	Palaeontology and archaeology
☒	☐	Animals and other organisms
☐	☒	Human research participants
☒	☐	Clinical data
☒	☐	Dual use research of concern

Methods

n/a	Involved in the study	
☒	☐	ChIP-seq
☒	☐	Flow cytometry
☐	☒	MRI-based neuroimaging

Eukaryotic cell lines

Policy information about cell lines

Cell line source(s)	1. UMUC3: source - ATCC; 2. LN-229: source - ATCC
Authentication	STR analysis at UC Berkeley Cell Culture Facility
Mycoplasma contamination	all cell lines were negative (mycoplasma testing - in-lab PCR testing)
Commonly misidentified lines (See ICLAC register)	No commonly misidentified lines were used in this study.

Human research participants

Policy information about studies involving human research participants

Population characteristics	The population includes participants of all ages and sex with a diagnosis of cancer.
Recruitment	Participants were recruited based on detection of the TERT duplication in the UCSF500 panel and GENIE data set. As patients

Recruitment	with certain tumor diagnosis or age are more likely to receive a panel sequencing of their tumor, certain diagnoses or age groups might be overrepresented in our cohort whereas others are underrepresented.
Ethics oversight	This study complies with all relevant ethical regulations and was approved by the Committee on Human Research of the University of California, San Francisco. All patients provided informed written consent prior to sample acquisition. Patients were not monetarily compensated.

Note that full information on the approval of the study protocol must also be provided in the manuscript.

Magnetic resonance imaging

Experimental design

Design type	Case report
Design specifications	Single patient report
Behavioral performance measures	NA

Acquisition

Imaging type(s)	Structural
Field strength	3T
Sequence & imaging parameters	T, post contrast sagittal and cranial planes (4a,4b)
Area of acquisition	Whole brain

Diffusion MRI ☐ Used ☒ Not used

Preprocessing

Preprocessing software	NA
Normalization	NA
Normalization template	NA
Noise and artifact removal	NA
Volume censoring	NA

Statistical modeling & inference

Model type and settings	NA
Effect(s) tested	NA

Specify type of analysis: ☒ Whole brain ☐ ROI-based ☐ Both

Statistic type for inference (See Eklund et al. 2016)	NA
Correction	NA

Models & analysis

n/a	Involved in the study
☒	☐ Functional and/or effective connectivity
☒	☐ Graph analysis
☒	☐ Multivariate modeling or predictive analysis
